# Supplementary material for: The receptor tyrosine kinase ErbB2/HER2 governs CDK4 inhibitor sensitivity, timing, and irreversibility of the G1/S transition
Source: J Biol Chem. 2025 Oct 27;301(12):110865. doi: 10.1016/j.jbc.2025.110865 (PMC12666559; doi:10.1016/j.jbc.2025.110865)
Supplement: Supporting Figures [file mmc1.pdf]

Supplementary information Figures for

The receptor tyrosine kinase ErbB2/HER2 governs CDK4 inhibitor sensitivity, timing and irreversibility of the G1/S transition

Fig. S1

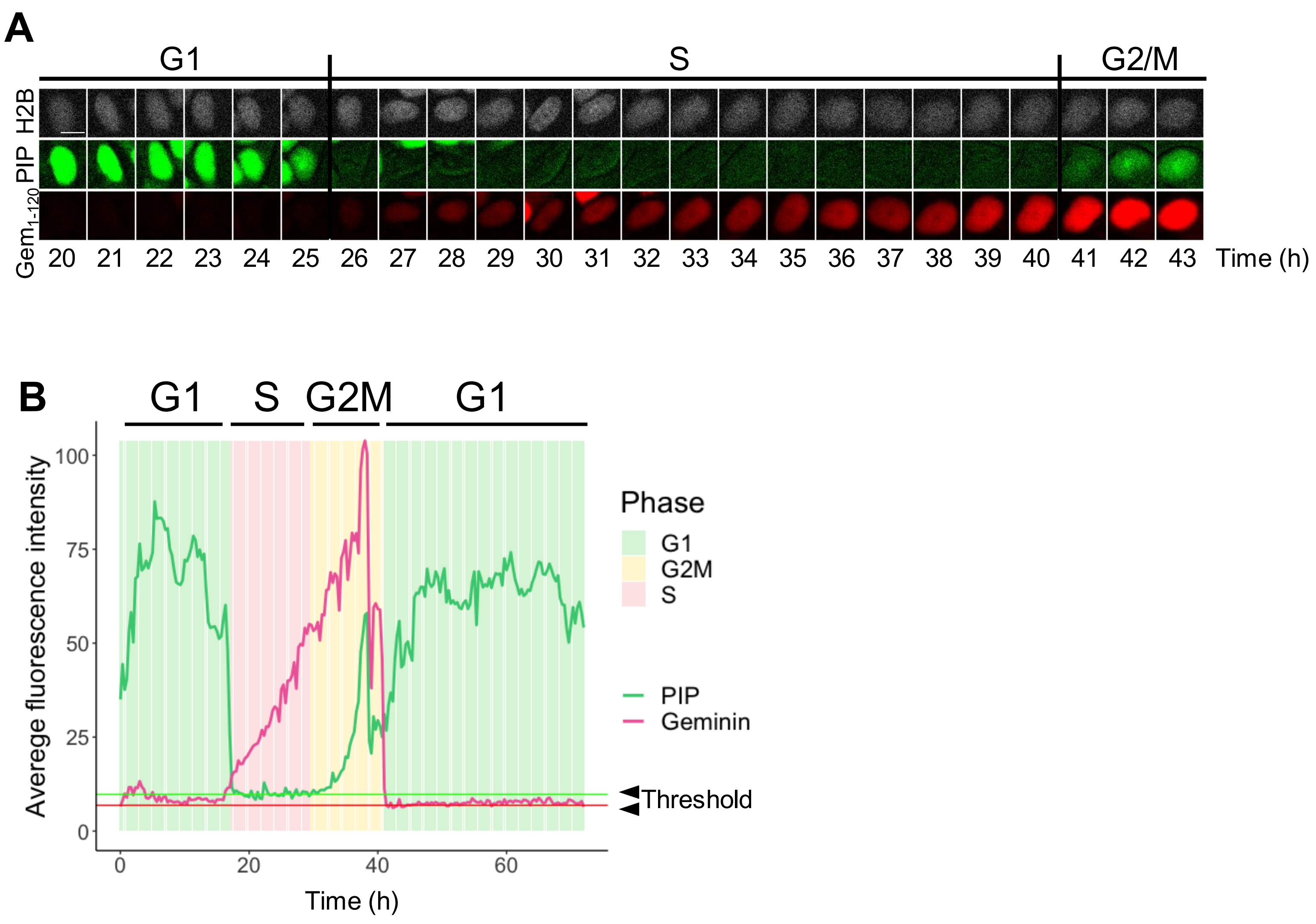

Fig. S1. Estimation of cell-cycle dynamics through single-cell tracking. (**A, B**) Data were acquired every 20 min for 72 h after 10 nM HRG stimulation in FUCCI stably expressing MCF-7 cells. (**A**) Representative examples of single-cell tracking with nuclear markers (H2B: gray), G1 and G2M markers (PIP-Cdt1: green), and S and G2M markers (red). (**B**) Cell cycle dynamics estimated below a set threshold from the variation in the average fluorescence intensity of PIP-Cdt1 and Geminin over time. For each cell cycle, green indicates the G1 phase, yellow indicates the G2M phase, and magenta indicates the S phase. The average fluorescence intensity of PIP-Cdt1 and Geminin is indicated by the green and magenta lines, respectively.

Fig. S2

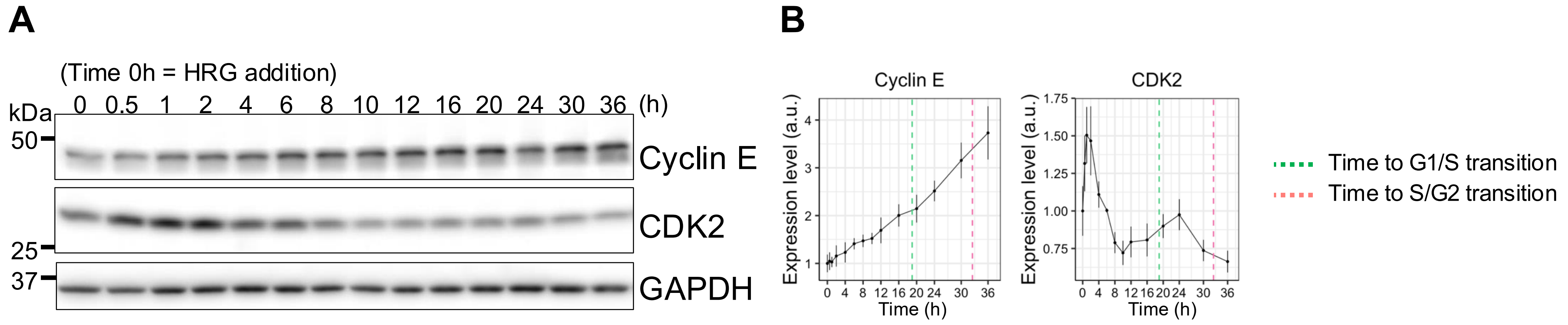

Fig. S2. Expression dynamics of signaling molecules and cell cycle regulators. (**A, B**) Expression of proteins regulating the G1/S transition in the ErbB pathway up to 36 h after 10 nM HRG treatment detected using western blotting. (**B**) Expression of each protein normalized relative to that of GAPDH and divided by that of the 0 h sample (0 h = 1). Graphs show means ± SEM from three independent replicates.

Fig. S3

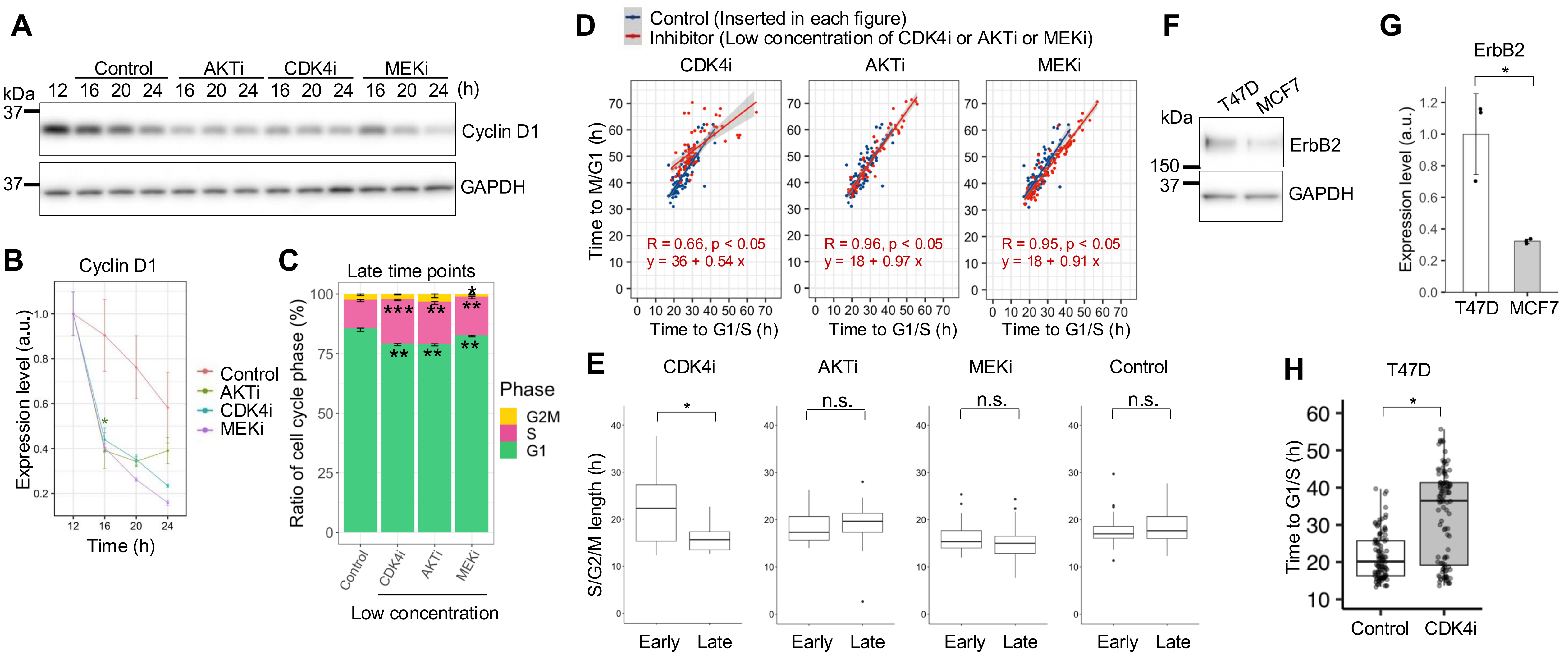

Fig. S3. Effect of CDK4, AKT, and MEK inhibitors on the expression of cell-cycle regulators and cell-cycle phase distribution. (A–B, E) FUCCI stably expressing MCF-7 cells were treated with each inhibitor (AKTi: 600 nM; CDK4i: 250 nM; MEKi: 500 nM; DMSO was used as the control) 12 h after 10 nM HRG stimulation. (A) Cells were harvested every 4 h until 24 h after 10 nM HRG stimulation, and expression of cyclin D1 and GAPDH was evaluated using western blotting. (B) Using the data from (A), the level of each protein was normalized to that of GAPDH and divided by that of the 12 h sample (12 h = 1). Graphs show means ± SEM for three independent experiments. (C) FUCCI stably expressing MCF-7 cells were treated with each inhibitor (AKTi: 200 nM; CDK4i: 100 nM; MEKi: 100 nM; DMSO was used as the control) 12 h after 10 nM HRG stimulation. Percentage distribution of each cell cycle phase (%) in the overall image at 72 h after HRG stimulation; n = 3. The data are displayed as the means ± SD. (D) Using the data from (C), data from cells that completed the M/G1 transition within 72 h. Control, n = 96; CDK4i, n = 74; AKTi, n = 60; MEKi, n = 86. Times to G1/S (x-axis) and M/G1 (y-axis) transitions, with slope (s) and intercept (i) obtained from the regression equation. (E) Images captured every 20 min until 72 h after 10 nM HRG stimulation were used to divide the population into an early and late subpopulation relative to the median time to the G1/S transition and to quantify the length of the S/G2/M phase in each population. CDK4i: n = 27, average time = 15.8 ± 2.8 h (late time), n = 25, average time = 22.3 ± 7.2 h (early time); AKTi: n = 50, average time = 18.4 ± 3.9 h (late time), n = 46, average time = 17.6 ± 2.9 h (early time); MEKi: n = 38, average time = 15.1 ± 3.6 h (late time), n = 37, average time = 16.0 ± 3.0 h (early time); Control: n = 21, average time = 18.9 ± 5.2 h (late time), n = 21, average time = 18.4 ± 3.6 h (early time). (F) Expression of ErbB2 and GAPDH determined using western blotting. (G) ErbB2 level normalized relative to that of GAPDH; n = 3. Graphs show means ± SD for three independent experiments. (H) Data from cells that completed the G1/S transition within 72 h. Control and CDK4i; n = 100. In (C) *P < 0.05, **P < 0.01, ***P < 0.001 (with respect to control and each inhibitor; two-tailed Student's *t*-test). In (B), (E), (G), and (H) *P < 0.05 (with respect to control and each inhibitor; Welch's *t*-test).

Fig. S4

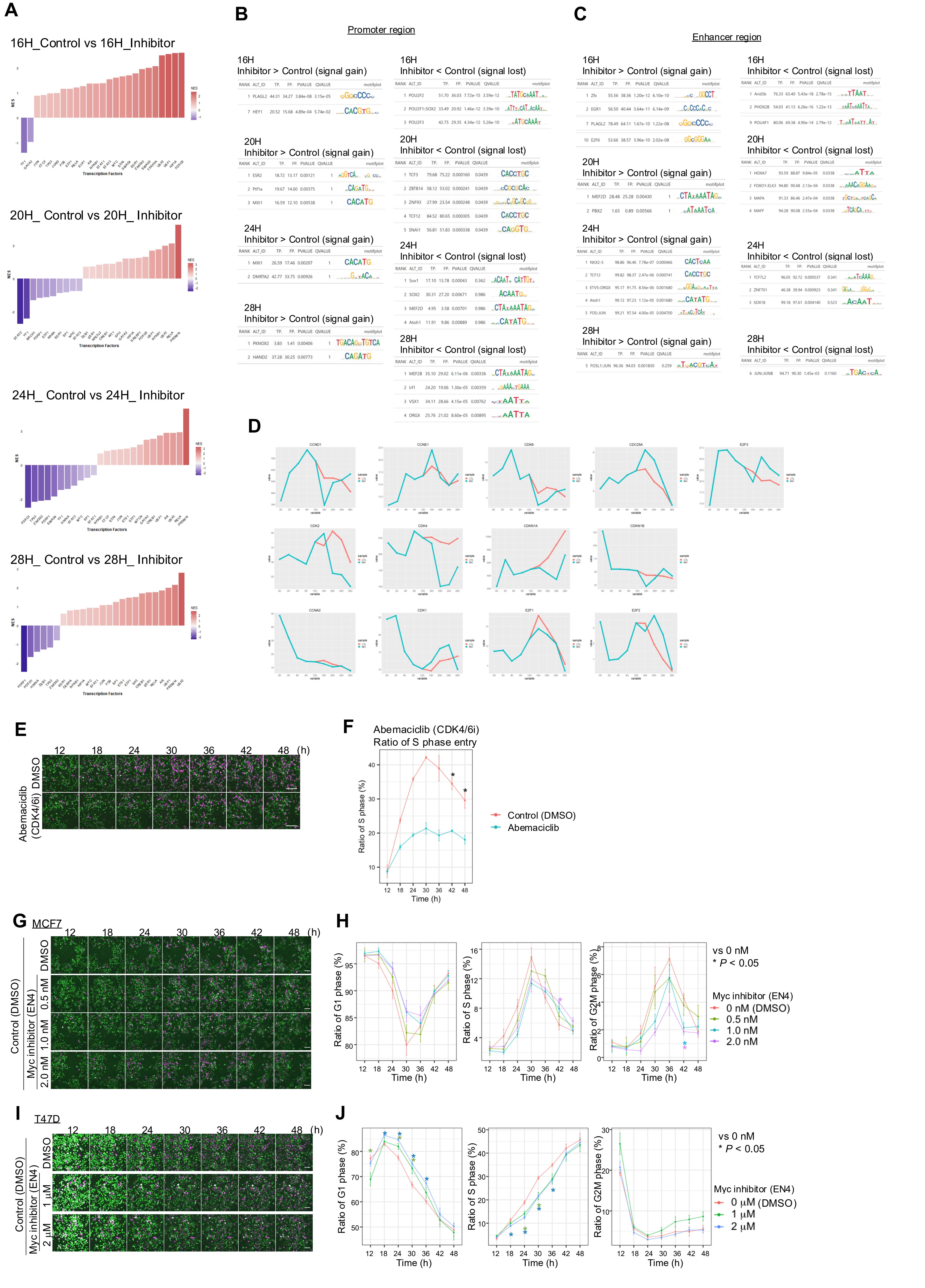
A
16H_Control vs 16H_Inhibitor
20H_ Control vs 20H_ Inhibitor
24H_ Control vs 24H_ Inhibitor
28H_ Control vs 28H_ Inhibitor
B
Promoter region
16H
Inhibitor > Control (signal gain)
16H
Inhibitor < Control (signal lost)
20H
Inhibitor > Control (signal gain)
20H
Inhibitor < Control (signal lost)
24H
Inhibitor > Control (signal gain)
24H
Inhibitor < Control (signal lost)
28H
Inhibitor > Control (signal gain)
28H
Inhibitor < Control (signal lost)
C
Enhancer region
16H
Inhibitor > Control (signal gain)
16H
Inhibitor < Control (signal lost)
20H
Inhibitor > Control (signal gain)
20H
Inhibitor < Control (signal lost)
24H
Inhibitor > Control (signal gain)
24H
Inhibitor < Control (signal lost)
28H
Inhibitor > Control (signal gain)
28H
Inhibitor < Control (signal lost)
D
E
12 18 24 30 36 42 48 (h)
DMSO
Abemaciclib (CDK4/6i)
F
Abemaciclib (CDK4/6i)
Ratio of S phase entry
Ratio of S phase (%)
Time (h)
Control (DMSO)
Abemaciclib
G
MCF7
12 18 24 30 36 42 48 (h)
Control (DMSO)
Myc inhibitor (EN4)
DMSO
0.5 nM
1.0 nM
2.0 nM
H
Ratio of G1 phase (%)
Ratio of S phase (%)
Ratio of G2M phase (%)
Time (h)
vs 0 nM
* P < 0.05
Myc inhibitor (EN4)
0 nM (DMSO)
0.5 nM
1.0 nM
2.0 nM
I
T47D
12 18 24 30 36 42 48 (h)
Control (DMSO)
Myc inhibitor (EN4)
DMSO
1 μM
2 μM
J
Ratio of G1 phase (%)
Ratio of S phase (%)
Ratio of G2M phase (%)
Time (h)
vs 0 nM
* P < 0.05
Myc inhibitor (EN4)
0 μM (DMSO)
1 μM
2 μM

Fig. S4. Transcriptomic and epigenetic analysis to detect transcriptional regulators associated with CDK4 inhibition. (A–C) FUCCI stably expressing MCF-7 cells were treated with 250 nM CDK4i or DMSO (control) 12 h after 10 nM HRG stimulation. Cells were then harvested every 4 h until 28 h after 10 nM HRG stimulation for RNA-seq and ChIP-seq of H3K27Ac and H3K4me1. (A) Transcription factor activity variation inferred using RNA-seq data under control and CDK4i conditions (log2 FC = CDK4i/control; DoRothEA). Blue and red indicate the decreased and increased activities of transcription factors, respectively. (B–C) Promoter (B) and enhancer regions (C) identified based on the H3K27Ac and H3K4me1 ChIP-seq peaks, respectively, and enrichment analysis was performed at each time point for the promoter/enhancer region. The signal under CDK4i conditions (whether gained or lost) was compared to that under control conditions. (D) Expression dynamics from 0 to 28 h after 10 nM HRG stimulation for cell cycle-related c-Myc-target genes. (E, F) After stimulation with 10 nM HRG for 12 h, WT MCF-7 were treated with 0 (DMSO) or 5 nM Abemaciclib. Scale bar: 200 µm. The percentage distribution of S phase over time was quantified (F). n = 3. The data are displayed as the means ± SEM. (G–J) After stimulation with 10 nM HRG for 11.5 h, MCF-7; n = 3 (G, H) and T47D; n = 6 (I, J) were treated with 0 (DMSO)/0.5/1.0/2.0 nM EN4 (G, H) or 0 (DMSO)/1/2 µM EN4 (I, J) for 30 min and then with DMSO. Scale bar: 100 µm. The percentage distribution of each cell cycle phase over time was quantified (H, J). The data are displayed as the means ± SEM. In (F), $^{*}P < 0.05$ (Comparison with the 30-hour time point for each condition after 30 hours; two-tailed Student's *t*-test). In (H), $^{*}P < 0.05$ (for control and each inhibitor condition; two-tailed Student's *t*-test). In (J), $^{*}P < 0.05$ (for control and each inhibitor condition; Welch's *t*-test).

Fig. S5

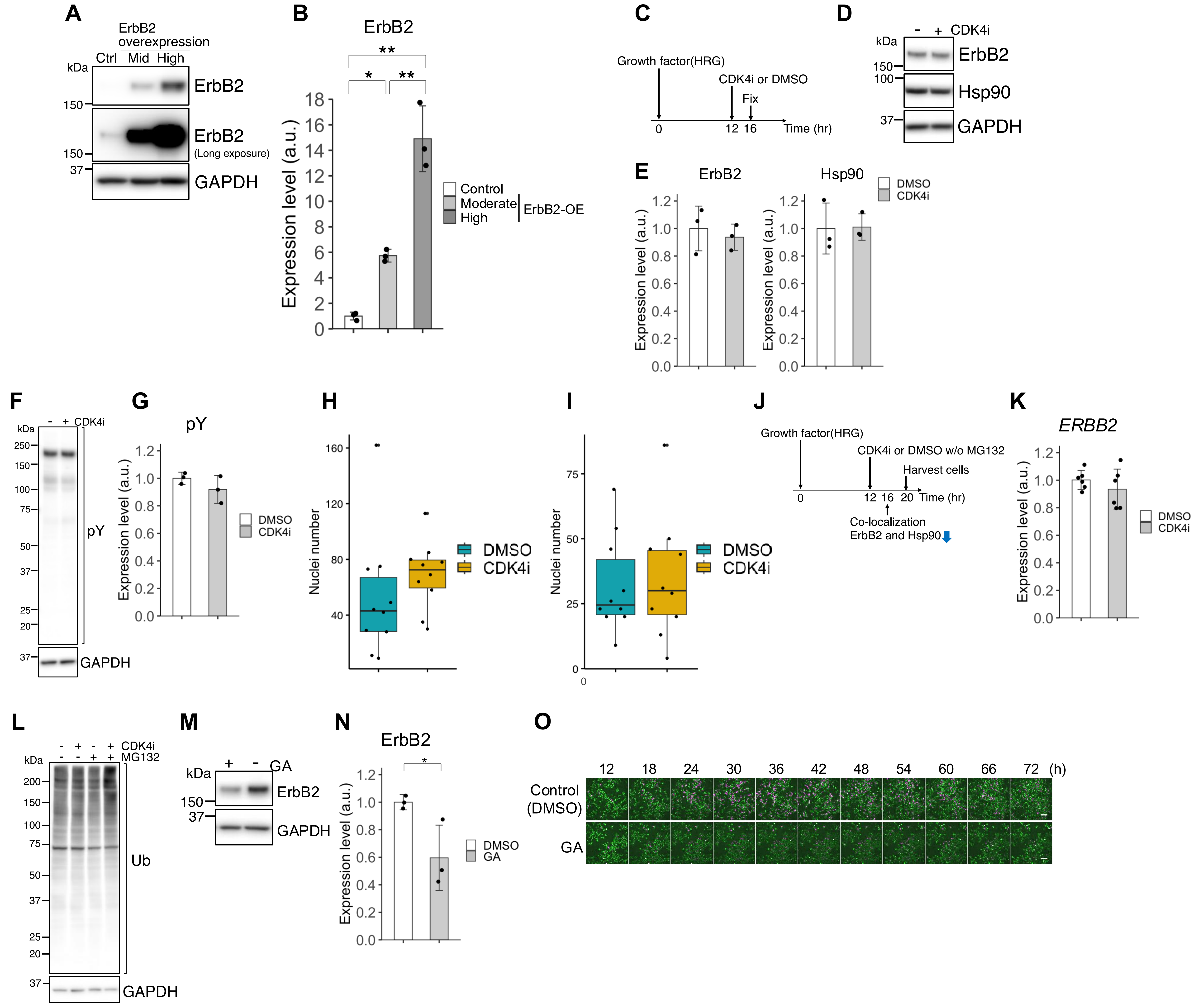

Fig. S5. CDK4 inhibition impairs c-Myc-mediated G1/S transition by reducing ErbB2 stabilization. (A, B) WT MCF-7 and ErbB2-overexpressing cells (moderate and high levels) were harvested 12 h after 10 nM HRG stimulation. (A) Expression of ErbB2 and GAPDH determined using western blotting. (B) ErbB2 relative to GAPDH; *n* = 3. The data are displayed as the means ± SD. (C–I) MCF-7 cells overexpressing ErbB2 (moderate levels) were independently treated with CDK4i or DMSO (control) 12 h after stimulation with 10 nM HRG and fixed or collected 4 h later. (C) Experimental scheme. (D) Expression of ErbB2, Hsp90, and GAPDH determined using western blotting. (E) ErbB2 and Hsp90 levels normalized relative to those of GAPDH; *n* = 3. The data are displayed as the means ± SD. (F) Expression of PY100 and GAPDH determined by western blotting. (G) PY100 relative to GAPDH, *n* = 3. The data are displayed as the means ± SD. (H, I) Number of nuclei from Fig. 5C (for H) and Fig. 5E (for I). (J–K) FUCCI stably expressing MCF-7 cells were treated with CDK4i or DMSO (control) as well as 10 μM MG132, 12 h after 10 nM HRG stimulation, and collected 8 h later. (J) Experimental scheme. (K) Comparison of ErbB2 mRNA expression detected using qPCR (n = 3 technical replicates with 2 biological replicates). The data are displayed as the means ± SD. (L) Ubiquitin detected using western blotting. (M, N) FUCCI stably expressing MCF-7 cells were harvested 8 h after being independently treated with 40 nM geldanamycin (GA) or DMSO (control), 12 h post 10 nM HRG stimulation. (M) Expression of ErbB2 and GAPDH determined using western blotting. (N) ErbB2 relative to GAPDH; *n* = 3. The data are displayed as the means ± SD. (O) 40 nM GA or DMSO (control) was used to treat the FUCCI stably expressing MCF-7 cells 12 h after HRG stimulation. Scale bar: 100 μm. In (B), *P < 0.05 (Tukey's multiple comparisons test). In (E), (G), (K), and (N), *P < 0.05 (two-tailed Student's t-test). In (H), and (I), *P < 0.05 (Welch's t-test).

Fig. S6

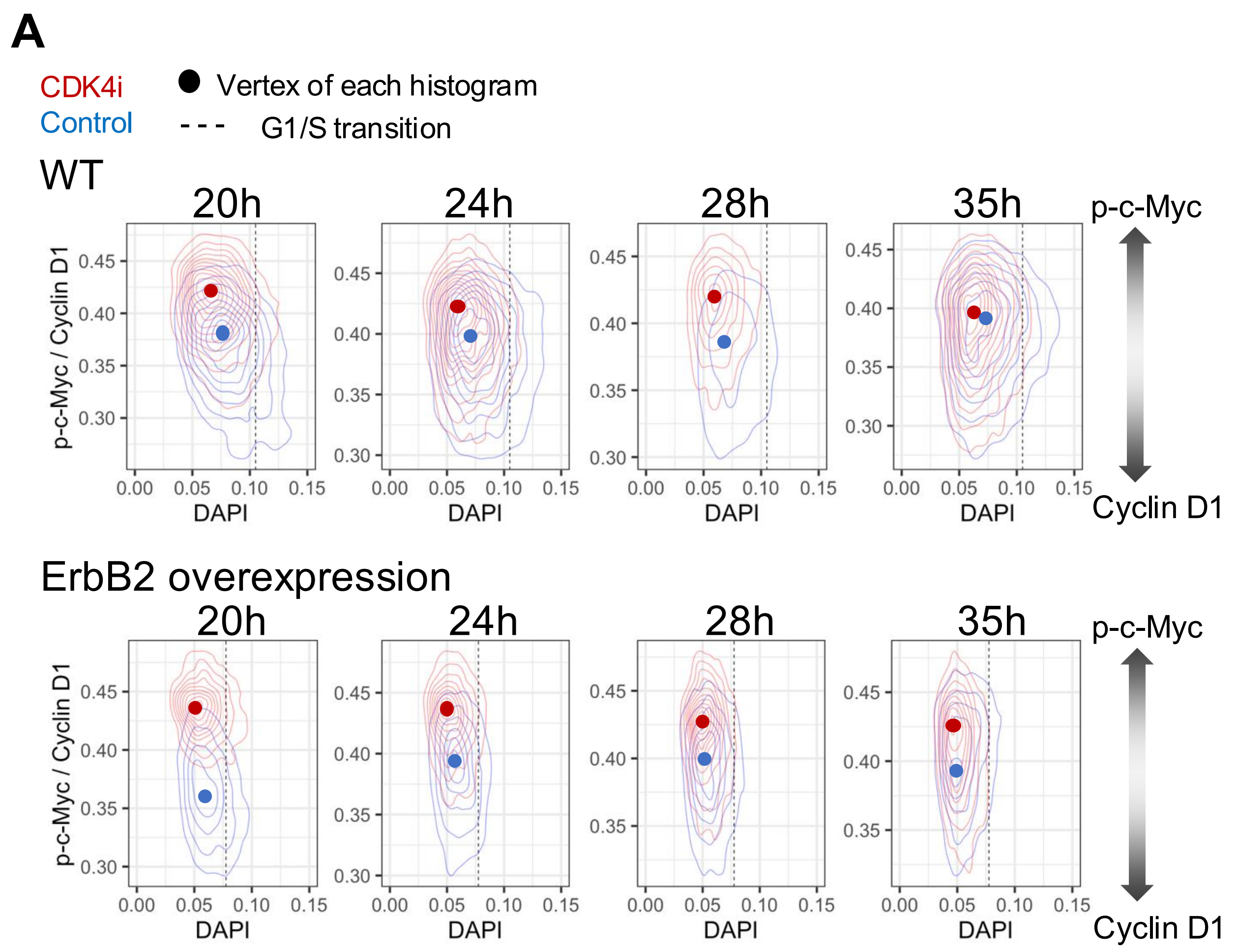

Fig. S6. p-c-Myc/cyclin D1 ratio is sustained in ErbB2-overexpressing cells compared to control cells. (**A**) Control and MCF-7 cells overexpressing ErbB2 were individually treated with 250 nM CDK4i or DMSO (control) 12 h after 10 nM HRG stimulation; 20, 24, 28, and 35 h after HRG stimulation, cells were fixed, and p-c-Myc and cyclin D1 were stained for quantification. The vertex of each histogram is indicated by a circle; the G1/S transition estimated from the DAPI staining intensity is indicated by a black dotted line. Blue indicates the control; red indicates the CDK4i condition; n = 3 420 for each condition.

MovieS1

MovieS1. 500 nM CDK4 inhibitor induces cell death. FUCCI stably expressing MCF-7 cells were treated with 500 nM CDK4i 12 h after 10 nM HRG stimulation. Scale bar: 100 μm

MovieS2

MovieS2. 50 nM Abemaciclib induces cell death. FUCCI stably expressing MCF-7 cells were treated with 50 nM Abemaciclib 12 h after 10 nM HRG stimulation. Scale bar: 100 µm
