# Supplementary material for: The receptor tyrosine kinase ErbB2/HER2 governs CDK4 inhibitor sensitivity, timing, and irreversibility of the G1/S transition
Source: J Biol Chem. 2025 Oct 27;301(12):110865. doi: 10.1016/j.jbc.2025.110865 (PMC12666559; doi:10.1016/j.jbc.2025.110865)
Supplement: Table S1 [file mmc2.docx]

**Table S1. The primers used for real-time PCR**

| Gene | Forward primer sequence (5'-3') | Reverse primer sequence (5'-3') |
| --- | --- | --- |
| *CDKN1B* | AGATGTCAAACGTGCGAGTG | TCTCTGCAGTGCTTCTCCAA |
| *ERBB2* | AACTGCACCCACTCCTGTGT | TGATGAGGATCCCAAAGACC |
| *RPL27* | CTGTCGTCAATAAGGATGTCT | CTTGTTCTTGCCTGTCTTGT |
| *PCNA* | CTGTAGCGGCGTTGTTGC | TCGTTGATGAGGTCCTTG |
